# Effects of Different Lactic Acid Bacterial Strains on the Physicochemical Properties and Flavor of Millet Fermented Beverages

**DOI:** 10.3390/foods15142491

**Published:** 2026-07-14

**Authors:** Yumeng Han, Chaofan Zhao, Yuting Zhu, Jiaxue Wang, Ruijia Yang, Wenting Wang, Shengyuan Guo, Runze Chen, Lizhen Zhang, Guixing Ren

**Affiliations:** 1 School of Life Science, Shanxi University, No. 92 Wucheng Road, Taiyuan 030006, China; 2 Environmental Science Research Institute, Shanxi University, Taiyuan 030006, China

**Keywords:** millet, fermented beverage, plant-based beverages, multi-strains, co-fermentation

## Abstract

To address the issues of rough mouthfeel and monotonous flavor in plant-based beverages, this study used extruded millet flour as the raw material, with non-inoculated millet paste as the control group, and investigated the effects of six different lactic acid bacteria starter culture formulations—including two single strains (*Streptococcus salivarius* subsp. *thermophilus* and *Lactobacillus delbrueckii* subsp. *bulgaricus*) and four multi-strain consortia (2, 4, 10, and 12 strains)—on the quality of fermented millet beverages. The structural properties of the fermented millet beverages were systematically evaluated through WHC (Water Holding Capacity), LF-NMR (Low-Field Nuclear Magnetic Resonance), RVA (Rapid Visco Analyzer), rheological properties, texture, particle size, and FTIR (Fourier Transform Infrared) analyses, covering aspects such as water status, pasting behavior, viscoelasticity, textural characteristics, particle distribution, and molecular structure. In combination with volatile flavor profiling and sensory evaluation, the fermentation performance and applicability of each starter formulation were comprehensively assessed. The results showed that MFB-2 exhibited the highest viscosity and gel strength, making it suitable for thick-set products, whereas MFB-10 and MFB-12 demonstrated superior gel stability, water-holding capacity, and shelf life. Sensory evaluation further corroborated the flavor analysis, with MFB-12 showing the best aroma and overall acceptability, alongside the greatest diversity of volatile compounds, while MFB-10 presented milder acidity and favorable sensory acceptability. Collectively, MFB-10 and MFB-12 were identified as the most promising starter culture formulations for industrial-scale production of fermented millet beverages. This study provides a scientific basis for tailoring starter culture complexity to modulate the texture and flavor of plant-based fermented products.

## 1. Introduction

Millets (*Setaria italica*) are a group of small-grain food crops. Studies have shown that millet is rich in dietary fiber, vitamins, minerals, iron, amino acids, fatty acids and plant compounds compared to major grains [1]. In addition, as a climate-resilient crop [2], millet can grow effectively on low-fertility marginal land and show significant adaptability to abiotic stresses such as drought. India accounts for approximately 26.6% of the global millet harvested area, with millets serving as a staple food for about 90 million people across Asia and Africa ss [3,4].

In recent years, the plant-based beverage market has grown fastest in the food industry as consumers’ concerns about healthy, environmentally friendly and sustainable diets have increased [5]. Plant-based beverages are based on raw materials such as grains, nuts, and beans [3], and are processed into beverage forms similar to dairy products [6]. Compared with animal-derived beverages, plant-based beverages have the advantages of low cholesterol, low saturated fat, rich dietary fiber and phytochemicals [4], aiming to replace or supplement dairy products [3]. However, traditional plant-based beverages often have bottlenecks such as rough taste, single flavor, poor protein stability and high content of anti-nutritional factor [7], which limit their consumption acceptance and nutritional value. Traditional cereal-based fermented beverages, such as boza (millet/maize-based, consumed in the Balkans) and ogi (fermented millet/sorghum in West Africa), have been consumed since the Neolithic period, yet their production remains largely artisanal with inconsistent quality and limited shelf life [3,8]. Therefore, improving the sensory quality and nutritional characteristics of plant-based beverages through microbial fermentation technology has become the frontier direction of current food science research [9]. As an important food processing technology, fermentation can effectively prolong the shelf life of food materials, enrich nutrients, optimize flavor and taste, enhance the biological activity of products, and comprehensively improve the original quality of food [10]. In the field of plant-based fermentation, lactic acid bacteria (LAB) are one of the core facets of fermentation [8]. The study [9] showed that the metabolism of lactic acid bacteria could reduce the pH value of the fermentation system, inhibit the growth of bacteria and improve the quality of plant protein. Unlike dairy fermentation, cereal-based fermentation faces distinct challenges due to the rigid globular structure of plant proteins, which limits protease accessibility, and the presence of complex oligosaccharides (e.g., raffinose-family oligosaccharides) that require specific enzymatic activities absent in most dairy-adapted starter cultures [8]. At present, the research on single-strain fermented cereal beverages has been relatively mature at home and abroad. For example, *Streptococcus salivarius* subsp. *thermophilus* or *Lactobacillus delbrueckii* subsp. *bulgaricus* are commonly used in yogurt products [11]. However, a single strain often has problems such as single metabolic capacity, insufficient flavor coordination, and poor adaptability in plant-based substrates. In contrast, multi-strain co-fermentation has been confirmed [12] to produce more complex metabolic networks through mutually beneficial symbiotic relationships between strains, thus showing significant advantages in the accumulation of flavor substances and the improvement of physical and chemical properties.

Although multi-strain fermentation has been widely used in dairy products, the research on plant-based beverages, especially millet fermented beverages, is still in its infancy. Most of the existing studies focus on a single strain or a simple combination of two strains, and there is still a lack of systematic comparative studies on the synergistic effect of higher complexity bacteria in the millet matrix. In particular, how the complexity of different strain combinations affects the structure and flavor characteristics of millet fermented beverages has not been fully elucidated.

Based on the above background, the study investigated the influence of strain synergistic effect on the physicochemical properties and flavor of the millet fermentation system to screen out the most suitable strain combination for the production of plant-based millet fermented beverage, including pH, water holding capacity, viscosity and other physical and chemical indicators during fermentation, and analyzing volatile flavor substances by gas chromatography–mass spectrometry (GC-MS). The study is expected to provide a theoretical basis and technical support for the development of plant-based fermented beverages with high quality and unique flavor.

## 2. Materials and Methods

### 2.1. Materials

Fermentation raw materials were extruded millet flour, purchased from Shangqiu Wufuzheng Food Co., Ltd. (Shangqiu, Henan, China). The contents of protein, fat and carbohydrate in extruded millet flour were 7.2, 2.1 and 77.7 g/100 g, respectively. *Lactobacillus delbrueckii* subsp. *bulgaricus* and *Streptococcus salivarius* subsp. *thermophilus* were purchased from Shaanxi Ruimao Biotechnology Co., Ltd. (Xi’an, Shaanxi, China). Multi-strains of lactic acid bacteria were bought from Angel Yeast Co., Ltd. (Yichang, Hubei, China) (2 bacterial types: maltodextrin, *Lactobacillus delbrueckii* subsp. *bulgaricus*, *Streptococcus salivarius* subsp. *thermophilus*; 4 bacterial types: maltodextrin, *Streptococcus salivarius* subsp. *thermophilus*, *Lactobacillus delbrueckii* subsp. *bulgaricus*, *Lactococcus lactis* subsp. *lactis*, *Bifidobacterium animalis* subsp. *lactis*; 10 bacterial types: maltodextrin, *Streptococcus salivarius* subsp. *thermophilus*, *Lactobacillus delbrueckii* subsp. *bulgaricus*, *Bifidobacterium longum* subsp. *longum*, *Bifidobacterium breve*, *Bifidobacterium animalis* subsp. *lactis*, *Bifidobacterium adolescentis*, *Bifidobacterium bifidum*, *Bifidobacterium longum* subsp. *infantis*, *Lactobacillus thermophilus*, *Lactobacillus reuteri*; 12 bacterial types: maltodextrin, *Streptococcus salivarius* subsp. *thermophilus*, *Lactobacillus delbrueckii* subsp. *bulgaricus*, *Bifidobacterium longum* subsp. *longum*, *Bifidobacterium animalis* subsp. *lactis*, *Bifidobacterium breve*, *Bifidobacterium longum* subsp. *infantis*, *Bifidobacterium adolescentis*, *Lactobacillus acidophilus*, *Lactobacillus rhamnosus*, *Lactobacillus reuteri*, *Lactobacillus plantarum*, *Lactobacillus casei*). The standards of lutein and β-carotene (purity ≥ 98%) were purchased from Beijing Solarbio Technology Co., Ltd. (Beijing, China). Other reagents used in the experiment were analytical grade.

### 2.2. Sample Preparation

The fermented millet beverages were prepared using extruded millet powder as the raw material, with six different lactic acid bacteria starter formulations. The extruded millet powder was first passed through a 100-mesh sieve. Then, 200 g of the sieved millet flour was mixed with 2 L of purified water, and the mixture was ground and homogenized using a colloid mill (50 LA colloid mill, Shanghai Donghua High-Pressure Homogenizer Factory, Shanghai, China). The obtained slurry was sterilized in a boiling water bath at 100 °C for 10 min and subsequently cooled to room temperature. Each lactic acid bacteria starter (2 g) was accurately weighed and directly mixed with 200 mL of the cooled millet slurry. Fermentation was carried out at 37 °C for 12 h, and the fermented products were stored at 4 °C. The resulting fermented millet beverages were designated as MFB-S, MFB-D, MFB-2, MFB-4, MFB-10, and MFB-12, corresponding to inoculation with *Streptococcus salivarius* subsp. *thermophilus*, *Lactobacillus delbrueckii* subsp. *bulgaricus*, and multi-strain consortia comprising 2, 4, 10, and 12 strains, respectively. The uninoculated millet paste served as the control group and was labeled MFB-0.

### 2.3. Composition Analysis

Nutritional components of fermented millet beverages were measured immediately after 12 h of fermentation in accordance with the standard methods of the American Association of Cereal Chemists (AACC, 2002). Specifically, fat content was determined using Method 44-15A, and protein content was quantified via Method 46-13 with a nitrogen-to-protein conversion factor of 6.25. Total starch content was determined using the total starch (α-amylase/glucoamylase, AA/AMG) assay kit (Megazyme International Ireland Ltd., Wicklow, Ireland) according to the manufacturer’s instructions. Briefly, 2 mL of the sample was mixed with 2 mL of cold 1.7 mol L^−1^ sodium hydroxide solution in an ice-water bath for 15 min. Then, 8.0 mL of sodium acetate buffer (0.6 mol L^−1^, pH 3.8) containing calcium chloride (0.005 mol L^−1^) was added, followed by the successive addition of 0.1 mL of AA and 0.1 mL of AMG (3300 U mL^−1^). After incubation at 50 °C for 30 min, 2.0 mL of the mixture was centrifuged at 13,000 rpm for 5 min. Subsequently, 1.0 mL of the supernatant was transferred to a microcentrifuge tube containing 4.0 mL of sodium acetate buffer (0.1 mol L^−1^, pH 5.0) with calcium chloride (0.005 mol L^−1^). Finally, 0.1 mL of this solution was transferred to a new microcentrifuge tube, and 3.0 mL of GOPOD reagent was added. The absorbance was measured at 510 nm after incubation at 50 °C for 5 min.

### 2.4. Determination of Total Carotenoids

Total carotenoid content of the fermented millet beverages was determined according to the method of [13] with slight modifications. Water-saturated n-butanol was prepared, and the mixture was allowed to stand at room temperature until stratification; the supernatant was then collected for use. Accurately weighed 0.6 g of sample was added to 6 mL of water-saturated n-butanol and extracted at room temperature for 3 h in the dark. The mixture was then centrifuged at 4 °C and 10,000× *g* for 10 min. The absorbance of the supernatant was measured at 450 nm. A standard curve was constructed using β-carotene concentrations as the *x*-axis and absorbance as the *y*-axis, yielding the regression equation Y = 0.0027X + 0.0472 (R^2^ = 0.9992). All procedures were performed under light-protected conditions, and each sample was analyzed in triplicate.

### 2.5. Water Holding Capacity (WHC) and pH

Water-holding capacity (WHC) of the fermented millet beverages was determined according to a modified method described previously [14]. A 20 g sample of the fermented beverage was weighed and recorded as m_2_, then transferred to a 50 mL centrifuge tube (pre-weighed as m_1_). The sample was centrifuged at 10,000× *g* and 4 °C for 15 min, and the supernatant was carefully removed. The centrifuge tube was then inverted for 30 min to drain residual liquid, after which it was immediately weighed and recorded as m_3_. WHC was calculated as follows:

$$\mathrm{WHC}\left(\%\right)=\left(\frac{m_{3}-m_{1}}{m_{2}}\right)\;\times \;100$$

where m_1_ is the weight of the centrifuge tube (g), m_2_ is the weight of the sample (g), and m_3_ is the total weight of the centrifuge tube and sediment after centrifugation (g).

The pH of the samples was measured at 24 °C using a pH meter (FiveEasy Plus, METTLER TOLEDO Instruments (Shanghai) Co., Ltd., Shanghai, China). Prior to measurement, the pH meter was calibrated with standard buffer solutions to ensure accuracy. Each sample was measured in triplicate, and the average value was taken as the final pH.

### 2.6. Low-Field Nuclear Magnetic Resonance (LF-NMR)

Freeze-dried fermented beverage samples (1 g) were placed in NMR tubes (diameter: 2.5 × 10^−4^ m), and a Carr-Purcell-Meiboom-Gill (CPMG) pulse sequence was applied using an LF-NMR analyzer (MESOMR23-060H-I, Suzhou Niumai Analytical Instruments Co., Ltd., Suzhou, China), following the method of [15] with slight modifications. The parameters were set as follows: waiting time = 3000 ms, echo time = 0.80000 ms, and echo number = 5000.

### 2.7. Pasting Properties

Starch pasting properties of the fermented millet beverages were determined using a rheometer (MCR 102e, Anton Paar, Graz, Austria). An 18 mL aliquot of the fermented beverage was placed in an aluminum container, and the mixture was stirred with a paddle rotor (ST24-2D/2V/2V-30) at 160 rpm. The temperature program was set as follows: holding at 50 °C for 1 min, heating to 95 °C at a rate of 12 °C/min, maintaining at 95 °C for 2.5 min, cooling to 50 °C at 12 °C/min, and finally holding at 50 °C for 2 min. Pasting parameters, including peak viscosity, trough viscosity, breakdown, final viscosity, and setback, were recorded from the resulting viscosity curve.

### 2.8. Rheological Properties

Rheological measurements were performed using an Anton Paar rotary rheometer (MCR 102e, Anton Paar GmbH, Graz, Austria) equipped with RheoCompass software (version 1.25, Anton Paar GmbH, Graz, Austria). The flow behavior of the samples was characterized by measuring the viscosity curve over a shear rate range of 0.1–100 s^−1^. Frequency sweep tests were conducted at angular frequencies from 0.1 to 100 rad/s. The experimental data were fitted using the Herschel–Bulkley model:

$$\tau =\tau_{0}+K\gamma^{n}$$

where τ is the shear stress (Pa), τ_0_ is the yield stress (Pa), K is the consistency coefficient (Pa·s^n^), and n is the flow behavior index (dimensionless). The storage modulus (G′) and loss modulus (G″) were also determined to evaluate the viscoelastic properties and internal structural stability of the samples under oscillatory shear conditions.

### 2.9. Texture of Fermented Millet Beverage

Texture properties of the fermented beverages were determined according to a previously described method [16], using a texture analyzer (TMS-Pro, FTC, Washington, DC, USA) equipped with a cylindrical probe (diameter: 36 mm). A 30 mL aliquot of each fermented millet beverage was placed in a 50 mL beaker, and a reverse extrusion test was performed at a test speed of 60 mm/min. Key texture parameters, including hardness (N), cohesiveness (ratio), springiness (mm), gumminess (N), and chewiness (mJ), were calculated using Texture Lab Pro software (Food Technology Corporation, Sterling, VA, USA).

### 2.10. Particle Size Analysis

Particle size distribution of the fermented millet beverages was measured using a nanoparticle size and zeta potential analyzer (BeNano 90 Zeta, Dandong Baite Instrument Co., Ltd., Dandong, Liaoning, China). The measurements were performed under the following parameters: dispersant refractive index = 1.33, equilibration time = 70 s, temperature = 25 °C, and scattering angle = 90°. Each sample was analyzed in triplicate.

### 2.11. Fourier Transform Infrared (FTIR) Spectroscopy

Fourier transform infrared (FTIR) spectra of the fermented millet beverages were recorded using a SPECTRUM 3 spectrometer (PerkinElmer, Waltham, MA, USA) equipped with a universal attenuated total reflectance (UATR) accessory. Each sample was placed onto a diamond/ZnSe crystal, and spectra were collected under standard ATR-FTIR conditions. The parameters were set as follows: wavenumber range of 650–4000 cm^−1^ and resolution of 4 cm^−1^. Spectral data in the amide I region (1600–1700 cm^−1^) were processed using PeakFit 4.12 software (SPSS Inc., Chicago, IL, USA).

### 2.12. GC-MS

Volatile compounds of the fermented millet beverages were analyzed using a gas chromatography–mass spectrometry (GC-MS) system (Trace1310 GC-ISQ7000 MS, Thermo Scientific, Waltham, MA, USA) equipped with a DB-WaxMS capillary column (30 m × 0.25 mm, 0.25 μm, Agilent, Santa Clara, CA, USA) and a DVB/CAR/PDMS solid-phase microextraction (SPME) fiber (Supelco, Bellefonte, PA, USA). The extraction procedure was based on a previously validated HS-SPME method for fermented beverages [17]. A 5.0 mL aliquot of each sample was transferred to a 20 mL headspace vial, and 5.0 μL of 2-octanol internal standard solution (10 mg/L) was added. The vial was immediately sealed, vortex-mixed for 30 s to ensure homogeneous dispersion of the internal standard, and then equilibrated in a water bath at 50 °C for 10 min. SPME extraction was performed using the DVB/CAR/PDMS fiber at 50 °C for 30 min. After extraction, the fiber was inserted into the GC inlet for thermal desorption at 250 °C for 5 min. All samples were analyzed in triplicate.

Retention indices (RI) of volatile compounds were calculated by analyzing a series of n-alkanes (C_8_–C_20_) under identical GC-MS conditions. Compound identification was performed by comparing the obtained mass spectra with the National Institute of Standards and Technology (NIST) library, and by matching the calculated retention indices with those reported in the literature (with deviations of RI < 50). Quantification was carried out using 2-octanol as the internal standard, and the relative content of each compound was determined by the peak area normalization method.

### 2.13. Sensory Analysis

Sensory evaluation was conducted using a nine-point hedonic scale. Fifteen panelists (nine females and six males) from the School of Life Science, Shanxi University, assessed the sensory attributes of the fermented beverage samples independently. Each sample was served to the panelists in disposable white plastic containers. Panelists were provided with water to rinse their palates between samples [14]. The samples were rated on a nine-point hedonic scale for appearance, taste, texture, and overall acceptability, where 1 = dislike extremely, 2 = dislike very much, 3 = dislike moderately, 4 = dislike slightly, 5 = neither like nor dislike, 6 = like slightly, 7 = like moderately, 8 = like very much, and 9 = like extremely [14].

### 2.14. Statistical Analysis

All experiments were performed in triplicate, and the results were expressed as mean ± standard deviation (SD). Statistical analysis was carried out using SPSS Statistics 23 (IBM SPSS Inc., Chicago, IL, USA). Differences among groups were assessed by one-way analysis of variance (ANOVA), followed by Duncan’s multiple range test for post hoc comparisons. A significance level of *p* < 0.05 was considered statistically significant. Figures were generated using Origin software (Origin 2026, OriginLab, Northampton, MA, USA).

## 3. Results and Discussion

### 3.1. Composition Analysis

#### 3.1.1. Protein

As shown in Table 1, the protein contents of MFB-4 (0.9589%) and MFB-10 (0.9531%) were the highest among all samples, and were significantly higher than that of the control group (*p* < 0.05). This may be attributed to the presence of *Lactococcus lactis* subsp. *lactis* in MFB-4, which possesses a cell envelope proteinase (CEP)-based proteolytic system [18]. Both MFB-4 and MFB-10 contained *Bifidobacterium*, which has been reported to utilize nitrogen sources such as small peptides to promote protein accumulation [19]. In contrast, the protein contents of MFB-2 and MFB-12 were significantly lower than that of MFB-0 (*p* < 0.05). This could be explained by the absence of *Bifidobacterium* in MFB-2, which may limit efficient nitrogen utilization [19], whereas MFB-12 contains *Lactobacillus rhamnosus* with known proteolytic capacity [20]. The lowest protein contents were observed in MFB-S and MFB-L, with no significant difference between them, suggesting that fermentation with *Lactobacillus delbrueckii* subsp. *bulgaricus* alone results in less protein accumulation than mixed-strain fermentation [21]. Overall, the protein content of the fermented beverages was influenced by the starter culture composition and their synergistic effects, with MFB-4 and MFB-10 exhibiting the highest levels, thus meeting the demand for high-protein products.

#### 3.1.2. Fat

As shown in Table 1, the fat contents in MFB-2, MFB-4, and MFB-10 were significantly lower than that of MFB-0, ranging from approximately 0.25% to 0.33%. This reduction is attributable to the lipase activity of both *Streptococcus salivarius* subsp. *thermophilus* and *Lactobacillus delbrueckii* subsp. *bulgaricus* [22,23]. However, compared with the synergistic metabolic effects of the two strains [24], the lipolytic capacity of a single strain is limited, and no significant difference in fat content was observed between the two single-strain groups, suggesting a similar degree of fat degradation, which was approximately 20% lower than that of MFB-0. Notably, although MFB-12 contained the highest number of bacterial types, its fat content did not decrease further; instead, it was higher than that of MFB-2, MFB-4, and MFB-10, and showed no significant difference from the single-strain groups, at approximately 0.51%. This may be explained by the presence of *Lactobacillus plantarum* in the MFB-12 starter culture, which has been reported to exert a strong inhibitory effect on lipase activity [25], thereby resulting in a relatively lower degree of fat hydrolysis. Overall, MFB-2, MFB-4, and MFB-10 exhibited the lowest fat contents among all fermented millet beverages, demonstrating the most effective fat-reducing performance, with no significant differences among these three groups.

#### 3.1.3. Total Starch

As shown in Table 1, no significant difference in total starch content was observed between MFB-S or MFB-L and the control group MFB-0. *Streptococcus salivarius* subsp. *thermophilus* cannot directly utilize macromolecular starch [26]. In contrast, the starch content of MFB-2 (69.5 mg/100 mL) was significantly higher than that of the single-strain groups, which may be attributed to the synergistic acid production of the multi-strain consortium, potentially leading to differential inhibition of amylase activity [27].

### 3.2. Total Carotenoid

Millet is rich in carotenoids, which are natural terpenoid pigments that play important roles in plant photosynthesis and photoprotection [13]. The color of millet is a key quality indicator, with deeper yellow hues generally associated with better sensory attributes.

As shown in Table 1, the carotenoid content of the control group (MFB-0) was 21.11 ± 0.37 μg/g, significantly higher than that of MFB-2, MFB-4, MFB-10, and MFB-12 (*p* < 0.05). During the initial thermal gelatinization step, carotenoids in MFB-0 underwent degradation reactions such as isomerization and oxidation, leading to reduced stability and content loss [28]. Among all samples, MFB-L exhibited the highest carotenoid content, suggesting that starter cultures containing *Lactobacillus delbrueckii* subsp. *bulgaricus* may favor carotenoid retention [29]. In contrast, all groups containing *Streptococcus salivarius* subsp. *thermophilus* showed low carotenoid levels, possibly due to the production of peroxidase (EfeB) during fermentation, which may contribute to carotenoid degradation.

In MFB-4, MFB-10, and MFB-12, carotenoid content decreased progressively with increasing microbial complexity. This trend may be attributed to the synergistic effect of acid production by *Lactobacillus delbrueckii* subsp. *bulgaricus* and the rapid metabolism of *Bifidobacterium*, which collectively lowered the pH of the fermentation system and promoted carotenoid degradation [30]. It is also possible that strain–strain interactions within complex metabolic networks trigger additional degradation mechanisms beyond the additive effects of individual strains. Consequently, MFB-L was identified as the most effective formulation for carotenoid retention. In contrast, all starter formulations containing *Streptococcus salivarius* subsp. *thermophilus*—whether as single or mixed strains—resulted in significant carotenoid loss and are therefore unsuitable for producing millet fermented beverages with high carotenoid content.

### 3.3. Water Holding Capacity (WHC)

Water-holding capacity (WHC) reflects the ability of a product to retain its own water, which directly influences sensory acceptability and shelf life [14]. As shown in Table 2, the WHC values of all fermented millet beverages generally exceeded 90%. Fermentation with starter cultures improved the lubrication characteristics and texture of the beverages [31]. MFB-12 and MFB-0 exhibited the highest WHC values. MFB-12 contained a variety of *Bifidobacterium* and *Lactobacillus plantarum* [32], suggesting that in situ exopolysaccharides (EPS) produced by *L. plantarum* may contribute to improved water-holding capacity. In contrast, MFB-2 showed significantly lower WHC than MFB-0 and MFB-12, and was the only sample with a marked reduction in WHC. According to a previous report [33], the pronounced pH drop in MFB-2 may have disrupted the protein network structure of the millet fermented beverage, thereby leading to its low WHC. The WHC values of MFB-S, MFB-L, MFB-4, and MFB-10 fell between those of the highest (MFB-0 and MFB-12) and lowest (MFB-2) groups, with no significant differences among them. Overall, MFB-12 demonstrated the best performance in terms of WHC, sensory texture, and shelf life, whereas MFB-2 exhibited the poorest water-holding capacity and is therefore not recommended as a preferred starter formulation. The remaining groups showed intermediate WHC levels with little variation among them.

### 3.4. LF-NMR

Low-field nuclear magnetic resonance (LF-NMR) was employed to determine the transverse relaxation time (T_2_) and the corresponding peak area ratios (A_2_) of water components in freeze-dried fermented millet beverage powders prepared with different starter cultures. The peak areas in LF-NMR relaxation analysis reflect the distribution of water populations [34]. Three distinct water fractions were identified based on relaxation times: T_21_ (0.1–1 ms), corresponding to tightly bound water; T_22_ (10–100 ms), corresponding to semi-bound water; and T_23_ (100–1000 ms), corresponding to free/flowing water [34]. As shown in Table 3, the observed relaxation times for the three fractions were 0.47–0.69 ms (T_21_), 11.02–60.23 ms (T_22_), and 110.24–284.36 ms (T_23_).

MFB-0 exhibited the highest proportion of bound water (81%), which may be attributed to heat treatment promoting the assembly of millet starch into a dense and stable three-dimensional gel network [35]. The T_21_ and A_21_ of MFB-S were significantly lower than those of the other groups (*p* < 0.05), indicating that its bound water was more tightly bound to the matrix but accounted for a lower proportion. This may be related to the rapid acidification and proteolytic capacity of *Streptococcus salivarius* subsp. *thermophilus* in the plant-based matrix [36], which may disrupt the protein network and reduce water-holding capacity. A shorter T_2_ suggests restricted molecular mobility, reflecting stronger binding between water and the surrounding matrix. MFB-L exhibited similar proportions of bound water (41%) and free water (39%). According to the literature [37], this may be because *Lactobacillus delbrueckii* subsp. *bulgaricus* has a slower acidification rate than *S. salivarius* subsp. *thermophilus*, along with proteolytic activity that causes comparatively less network damage. MFB-2 showed a relatively high bound water proportion (56%), but still contained 28% free water. This may be attributed to the rapid synergistic acidification of the two traditional starter strains, as the pH decrease during acid-induced gelation leads to protein network contraction and a significant reduction in WHC [33]. The T_22_ proportion in MFB-4 increased to 22.44%, which may be due to the introduction of *Bifidobacterium animalis* subsp. *lactis*, enabling co-fermentation with *S. salivarius* subsp. *thermophilus* [38] and promoting EPS production, thereby improving textural stability [39]. MFB-10 exhibited a remarkably high bound water proportion (72%) and extremely low free water (only 6%), which is consistent with the high EPS-producing characteristics of its multi-strain system. A previous study [32] confirmed that EPS production by high-yield EPS lactic acid bacteria starters was significantly positively correlated with WHC and A_22_ (*p* < 0.05), and significantly negatively correlated with A_23_. MFB-12, which contained the highest number of bacterial types, showed A_21_ of 46.94%, A_22_ of 31.73%, and A_23_ of 21.34%, indicating an ideal gel network structure. According to existing reports [40], this may be due to the synergistic production of EPS with diverse structures and complementary rheological properties by the multiple strains in MFB-12.

In summary, MFB-12 and MFB-10 exhibited the best water retention and gel network structure, making them the most suitable starter formulations for producing high-quality fermented millet beverages.

**Table 3:** The transverse relaxation time (T_2_) and peak integral area (A_2_) of millet fermented beverage freeze-dried powder inoculated with different lactic acid bacteria starters.

Sample	Transverse Relaxation Time			Peak Integral Ratio		
	T_21_ (ms)	T_22_ (ms)	T_23_ (ms)	A_21_ (%)	A_22_ (%)	A_23_ (%)
MFB-0	0.67 ± 0.03 a	41.21 ± 8.61 b	162.93 ± 13.35 c	81.07 ± 0.92 a	9.57 ± 1.45 c	9.36 ± 1.21 e
MFB-S	0.47 ± 0.11 b	11.02 ± 2.30 d	110.24 ± 13.68 d	28.57 ± 10.79 d	20.43 ± 5.24 b	51.00 ± 6.90 a
MFB-L	0.68 ± 0.07 a	21.64 ± 5.32 c	167.16 ± 19.36 c	41.05 ± 5.31 c	19.85 ± 3.66 b	39.10 ± 5.53 b
MFB-2	0.62 ± 0.13 ab	25.15 ± 3.83 c	148.76 ± 16.01 c	56.22 ± 2.95 b	16.13 ± 1.17 bc	27.65 ± 2.48 cd
MFB-4	0.69 ± 0.18 a	29.47 ± 3.66 c	163.76 ± 24.30 c	47.43 ± 4.38 bc	22.44 ± 6.31 b	30.13 ± 1.93 c
MFB-10	0.61 ± 0.02 ab	60.23 ± 4.71 a	284.36 ± 29.62 a	72.44 ± 1.10 a	21.30 ± 2.42 b	6.26 ± 1.67 e
MFB-12	0.69 ± 0.05 a	46.62 ± 1.85 b	219.99 ± 15.26 b	46.94 ± 5.00 bc	31.73 ± 2.94 a	21.34 ± 2.52 d

Note: Values are means ± STD (n = 3). Values with different letters in the same column are significantly different (*p* < 0.05). MFB-0, MFB-S, MFB-L, MFB-2, MFB-4, MFB-10, and MFB-12 represent millet fermented beverage samples inoculated with *Streptococcus salivarius* subsp. *thermophilus*, *Lactobacillus delbrueckii* subsp. *bulgaricus*, 2 strains, 4 strains, 10 strains and 12 strains, respectively.

### 3.5. PH

The pH values of the fermented millet beverages are presented in Table 2. Compared with the control group (MFB-0), all starter culture formulations exhibited varying degrees of acidification capacity. MFB-S (3.91 ± 0.05) showed a lower pH than MFB-L (4.03 ± 0.02), which may be attributed to the higher acidification ability of *Streptococcus salivarius* subsp. *thermophilus* compared with *Lactobacillus delbrueckii* subsp. *bulgaricus* [37]. The two-strain starter (MFB-2) displayed stronger acidification than either single-strain culture [38], while MFB-4, MFB-10, and MFB-12 exhibited considerably greater acid-producing capacity than both single- and two-strain starters. This enhancement is likely due to the synergistic effects of mixed starter cultures, which significantly improved fermentation efficiency [41]. Among all samples, MFB-4 demonstrated the strongest acidification, consistent with the notion that co-fermentation with four strains increases bacterial population and metabolite abundance, thereby promoting acid production [42]. MFB-10 and MFB-12 also showed pronounced acidification, although their pH values were slightly higher than that of MFB-4. This may be explained by the fact that starter cultures containing *Bifidobacterium* tend to result in fermented products with relatively higher pH [43]. In summary, MFB-4 exhibited the highest acidity among all tested formulations, followed by MFB-10 and MFB-12.

### 3.6. Pasting Properties

**Figure 1:**
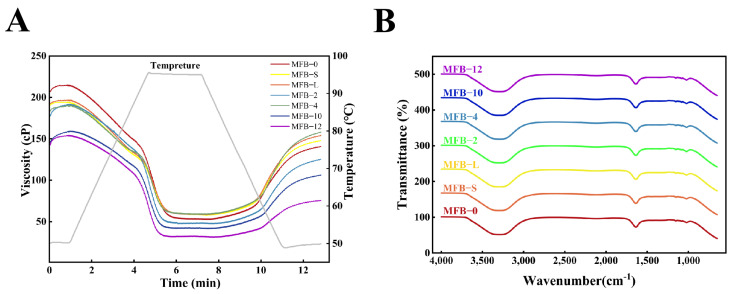
RVA (**A**) and FTIR (**B**) analysis results of millet fermented beverages inoculated with different lactic acid bacteria starters. RVA: rapid viscosity analyzer. FTIR: Fourier transform infrared. MFB-0, MFB-S, MFB-L, MFB-2, MFB-4, MFB-10, and MFB-12 represent millet fermented beverage samples inoculated with *Streptococcus salivarius* subsp. *thermophilus*, *Lactobacillus delbrueckii* subsp. *bulgaricus*, 2 strains, 4 strains, 10 strains and 12 strains, respectively.

The pasting properties of millet fermented beverages inoculated with different starter cultures were determined using a Rapid Visco Analyzer (RVA), and the gelatinization curves are presented in Figure 1A. Heat treatment induces gelatinization of millet starch, during which amylose and amylopectin leach out from swollen granules and reassociate upon cooling to form a three-dimensional gel network with enhanced stability [35]. As shown in Table 4, MFB-12 exhibited the lowest values for peak viscosity, trough viscosity, breakdown, final viscosity, and setback among all samples.

Compared with MFB-0, the peak viscosity decreased significantly as the number of starter culture strains increased, indicating that fermentation reduced the peak viscosity of millet starch. This may be attributed to the high starch content and low protein interference in MFB-0, which allowed starch granules to fully swell upon heating, resulting in higher peak viscosity, trough viscosity, breakdown, final viscosity, and setback [44]. Following acidic fermentation, the surface of starch granules was eroded, forming depressions, irregular pores, and large holes [45]. The increased availability of action sites for amylase produced by lactic acid bacteria disrupted the starch granule structure, rendering the granules more susceptible to water absorption and swelling during gelatinization [45]. A previous study [45] also showed that reduced protein content in rice flour increased its peak viscosity. Breakdown viscosity, which reflects starch thermal tolerance, was lower in all fermentation groups than in MFB-0 and decreased with increasing bacterial complexity, suggesting that fermentation improved the thermal stability and shear resistance of starch, making it less prone to rupture under high temperatures [46].

The reduction in pasting parameters observed in samples with lower pH (MFB-4, MFB-10, and MFB-12) can be attributed to acid hydrolysis during fermentation. As reported by Putri et al. [47], lactic acid fermentation causes depolymerization of starch structure and weakens granule organization, resulting in lower peak viscosity. Similarly, acid hydrolysis has been shown to significantly reduce peak, final, and setback viscosities of starch [48,49]. This is mechanistically explained by the reduction in starch molecular weight, which facilitates the breakdown of swollen granules during pasting and weakens gel network formation upon cooling [50]. The setback value, an indicator of starch retrogradation [46], was significantly higher in MFB-0 (85.35 cP) than in MFB-2, MFB-10, and MFB-12 (45.86–76.90 cP), indicating that moderate fermentation effectively inhibited starch retrogradation and thereby delayed staling, thus improving shelf stability. As shown in Figure 1A, an increase in pasting parameters was observed upon cooling, which is attributed to the setback phenomenon resulting from the reassociation of amylose molecules during the cooling phase [35].

Based on a comprehensive evaluation of starch viscosity, thermal stability, and shelf life, MFB-10 and MFB-12 exhibited the best overall performance. They not only reduced peak viscosity and improved thermal stability but also effectively inhibited starch setback, thereby promoting stable beverage texture and extended shelf life.

### 3.7. Rheological Properties

#### 3.7.1. Stable Rheological Properties

**Figure 2:**
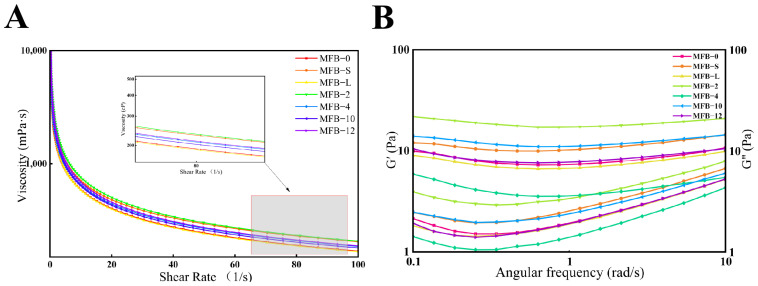
Stable rheological curve (**A**) and dynamic rheological curve (**B**) analysis results of millet fermented beverages inoculated with different lactic acid bacteria starters. MFB-0, MFB-S, MFB-L, MFB-2, MFB-4, MFB-10, and MFB-12 represent millet fermented beverage samples inoculated with *Streptococcus salivarius* subsp. *thermophilus*, *Lactobacillus delbrueckii* subsp. *bulgaricus*, 2 strains, 4 strains, 10 strains and 12 strains, respectively.

Rheology provides a key optimization basis for food process design by studying the flow and deformation behavior of materials [51]. Viscosity is an important quality parameter that affects the sensory characteristics of fermented beverage products [52]. The steady-state rheological properties of millet fermented beverages inoculated with different starter cultures were analyzed. As shown in Figure 2A, the viscosity of all samples decreased significantly with increasing shear rate, indicating non-Newtonian shear-thinning behavior, which is influenced by both the processing method and the starter culture type [53]. This shear-thinning behavior can be attributed to the progressive disruption of intermolecular associations within the starch gel network as shear force increases, leading to reduced flow resistance. Heat treatment induces starch gelatinization, during which amylose and amylopectin leach out from swollen granules and reassociate upon cooling to form a three-dimensional gel network [35]. Additionally, exopolysaccharides (EPS) synthesized by lactic acid bacteria can improve the mouthfeel of fermented products and serve as natural thickening and stabilizing agents, enhancing texture stability and shelf life [39].

As shown in Figure 2A, when the shear rate approaches zero, all samples exhibit high apparent viscosity, indicating a well-developed starch network under static conditions [35]. The sharp decrease in viscosity with increasing shear rate results from the disruption of network crosslinks and the alignment of polymer chains along the flow direction. All samples exhibit non-Newtonian shear-thinning behavior [44]. The breakdown of the gel network under increasing shear force resulted in a two-stage viscosity reduction: an initial sharp decline followed by a gradual decrease and eventual leveling off. Notably, the viscosity curves of all groups did not overlap across the entire shear rate range, suggesting that the effect of the starter culture persists throughout. The relatively high viscosity of the control group (MFB-0) can be attributed primarily to its higher starch content and lower protein interference, which allowed more extensive swelling of starch granules and greater leaching of amylose during gelatinization, thereby forming a stronger gel network upon cooling [44]. The inset magnification shows that the curves of each group maintained significant differences at medium to high shear rates, indicating that rheological differences between samples persisted even under strong shear, albeit with diminished magnitude, which may be related to the differential effects of starter cultures on protein conformation and starch–protein interactions. Among all samples, MFB-2 consistently exhibited the highest viscosity, indicating the greatest flow resistance. This is likely because the co-culture of the two strains shortens the lag phase and accelerates the growth rate compared with single-strain fermentation, resulting in slightly higher acidification capacity and improved growth performance [11]. In summary, MFB-2 exhibits optimal rheological properties owing to the synergistic effect of the two bacterial strains, meeting the requirements for a high-quality fermented millet beverage.

#### 3.7.2. Dynamic Rheological Properties

To investigate the dynamic rheological properties of the samples, frequency sweep tests were performed on millet fermented beverages prepared with different starter cultures. This approach effectively characterizes the variation in viscoelasticity as a function of time scale, yielding two key rheological parameters: the storage modulus (G′) and the loss modulus (G″) [51]. The frequency sweep results are presented in Figure 2B. All samples exhibited elastic-dominant behavior (G′ > G″) across the angular frequency range of 0.1–10 rad/s, with G′ showing little frequency dependence, indicating that the systems consistently maintained a solid-like gel structure without transitioning to a liquid-like state [54]. In the gel state, viscoelastic properties depend on the network formed by transient bonds. The frequency-independent storage modulus is characteristic of a well-structured gel with relatively low mechanical loss [55]. The system was primarily governed by elastic contributions, forming a stable three-dimensional network. Depending on the starter culture type, the elastic strength exhibited a clear gradient: MFB-2 showed the highest G′ and the densest network, whereas MFB-0 exhibited the lowest G′ and weaker structural stability. In the high-frequency region, G″ of MFB-4 increased rapidly, indicating enhanced viscous response, although the elastic network was still maintained. Overall, the starter culture type significantly modulated gel network strength. MFB-2 exhibited the highest gel strength and the densest network, making it the optimal formulation for texture and mouthfeel among the fermented millet beverages.

### 3.8. Texture of Fermented Millet Beverage

The rheological properties of fermented milk determine its texture and mouthfeel, and the complex structure is sensitive to temperature and shear forces [56]. The structure and texture of fermented millet beverages are primarily established during heat treatment [35]. Fermented millet beverages produced with starter cultures exhibiting high EPS production demonstrated improved lubrication characteristics and texture. The acidification rate of *Streptococcus salivarius* subsp. *thermophilus* also influences gel formation and the final texture of the fermented product [31]. As shown in Table 5, the hardness of MFB-S and MFB-10 was significantly higher than that of the control group. This may be attributed to the high acidification rate and associated proteolytic activity of *S. salivarius* subsp. *thermophilus*, which are known to contribute to a firm gel structure in fermented products [57]. The synergistic effect of multiple strains of streptococci and lactic acid bacteria accelerated acid production and enhanced the stability of the protein network [58]. In contrast, MFB-12 exhibited a significant reduction in hardness, which may be due to the pronounced proteolytic activity of *Lactobacillus rhamnosus*. Following fermentation, various organic acid metabolites—including phenylacetic acid and 3-phenyllactic acid—were produced, which moderately degraded the protein network and increased product fluidity [59]. Cohesiveness is another important texture parameter for yogurt-like products [56]. MFB-2 exhibited the highest cohesiveness, likely due to the synergistic acidification and proteolysis mechanisms of traditional *Lactobacillus delbrueckii* subsp. *bulgaricus* and *S. salivarius* subsp. *thermophilus*, which promote casein gel formation and enhance structural stability [58]. Metabolomics analysis [58] indicated that the protein network of dual-strain fermentation was superior to that of single-strain fermentation due to symbiotic effects; accordingly, the cohesiveness of multi-strain co-fermented samples was generally higher than that of single-strain samples. Gumminess, which is closely related to consumer acceptance, is another important texture parameter for yogurt [56]. MFB-S and MFB-10 showed significantly higher gumminess values than the control group (*p* < 0.05), with a more pronounced chewing sensation compared with hardness. The gumminess and chewiness of the remaining samples did not differ significantly from those of the control group. Among all samples, MFB-12 exhibited the lowest chewiness (4.14 mJ), indicating a smoother and more lubricated mouthfeel.

In summary, MFB-10 and MFB-12 demonstrated a well-balanced overall texture, making them suitable for industrial production. MFB-2 exhibited a firm and thick texture, which may appeal to consumers preferring a solid-type beverage.

**Table 5:** Texture profile analysis of millet fermented beverages inoculated with different lactic acid bacteria starters.

Sample	Hardness (N)	Cohesiveness (Ratio)	Springiness (mm)	Gumminess (N)	Chewiness (mJ)
MFB-0	0.55 ± 0.04 b	0.81 ± 0.03 bc	9.59 ± 0.53 b	0.44 ± 0.05 b	4.25 ± 0.72 b
MFB-S	0.77 ± 0.04 a	0.79 ± 0.01 c	10.36 ± 0.25 a	0.61 ± 0.04 a	6.32 ± 0.51 a
MFB-L	0.57 ± 0.04 b	0.78 ± 0.01 c	9.87 ± 0.33 ab	0.45 ± 0.03 b	4.43 ± 0.40 b
MFB-2	0.50 ± 0.04 b	0.86 ± 0.04 a	10.00 ± 0.40 ab	0.43 ± 0.02 b	4.28 ± 0.31 b
MFB-4	0.57 ± 0.04 b	0.82 ± 0.00 abc	9.87 ± 0.39 ab	0.47 ± 0.03 b	4.64 ± 0.51 b
MFB-10	0.70 ± 0.09 a	0.84 ± 0.02 ab	10.43 ± 0.07 a	0.58 ± 0.08 a	6.09 ± 0.84 a
MFB-12	0.50 ± 0.04 b	0.83 ± 0.02 ab	9.83 ± 0.30 ab	0.42 ± 0.03 b	4.14 ± 0.32 b

Note: Values are means ± STD (*n* = 3). Values with different letters in the same column are significantly different (*p* < 0.05). MFB-0, MFB-S, MFB-L, MFB-2, MFB-4, MFB-10, and MFB-12 represent millet fermented beverage samples inoculated with *Streptococcus salivarius* subsp. *thermophilus*, *Lactobacillus delbrueckii* subsp. *bulgaricus*, 2 strains, 4 strains, 10 strains and 12 strains, respectively.

### 3.9. Particle Size and Polydispersity Index

The Z-average particle size and polydispersity index (PDI) of millet fermented beverages inoculated with different starter cultures are presented in Table 6. The particle size followed the order: MFB-S > MFB-2 ≈ MFB-10 > MFB-0 > MFB-L ≈ MFB-4 > MFB-12, with significant differences between most groups (*p* < 0.05). The PDI values of all samples were ≤0.17, indicating a relatively narrow particle size distribution, as PDI < 0.2 is generally considered to reflect good monodispersity [60]. Among them, MFB-S and MFB-4 exhibited the lowest PDI (both 0.06), whereas MFB-2 showed a relatively higher PDI (0.17), suggesting slightly inferior particle size uniformity.

The use of *Streptococcus salivarius* subsp. *thermophilus* alone in MFB-S resulted in a significant increase in particle size, which is primarily attributed to its high-yield exopolysaccharide (EPS) production. EPS can promote protein aggregation or the formation of macromolecular networks, thereby increasing particle diameter [61]. In contrast, MFB-L exhibited a very small particle size. A previous study [62] reported that *Lactobacillus delbrueckii* subsp. *bulgaricus* does not produce EPS when fermented alone and may instead depolymerize starch molecules, potentially leading to reduced particle size. MFB-2 showed an intermediate particle size between MFB-S and MFB-L, suggesting that both aggregation [61] and depolymerization [62] may occur simultaneously within the system. The relatively higher PDI (0.17) of MFB-2 may indicate the presence of aggregated particle subsets of varying sizes.

The smallest particle size observed in MFB-12 is particularly noteworthy. This may be attributed to the strong proteolytic activity of *Lactobacillus plantarum*, which disrupts the protein network [63], as well as the cell envelope proteinases (CEPs) of *Lactobacillus rhamnosus* and *Lactobacillus casei*, which cleave protein peptide bonds [64]. In summary, the bacterial composition substantially influences particle size; MFB-12 produced smaller and more uniform particles, offering a potential strategy for modulating texture and mouthfeel in fermented millet beverages.

**Table 6:** The Z-average particle size and PDI value of the freeze-dried powder of millet fermented beverages inoculated with different lactic acid bacteria starters.

	Z-Average Particle Size (nm)	PDI
MFB-0	1536.97 ± 36.09 c	0.09 ± 0.07 ab
MFB-S	2193.27 ± 57.11 a	0.06 ± 0.04 b
MFB-L	1352.29 ± 28.38 d	0.10 ± 0.06 ab
MFB-2	1765.99 ± 63.70 b	0.17 ± 0.02 a
MFB-4	1435.80 ± 28.54 d	0.06 ± 0.02 a
MFB-10	1751.88 ± 80.15 b	0.10 ± 0.03 ab
MFB-12	1172.33 ± 76.72 e	0.14 ± 0.04 ab

Note: Values are means ± STD (*n* = 3). Values with different letters in the same column are significantly different (*p* < 0.05). PDI: Polydispersity Index. MFB-0, MFB-S, MFB-L, MFB-2, MFB-4, MFB-10, and MFB-12 represent millet fermented beverage samples inoculated with *Streptococcus salivarius* subsp. *thermophilus*, *Lactobacillus delbrueckii* subsp. *bulgaricus*, 2 strains, 4 strains, 10 strains and 12 strains, respectively.

### 3.10. FTIR Analysis

Fourier transform infrared (FTIR) spectroscopy is a highly sensitive technique used to analyze the secondary structures of starch, polysaccharides, and proteins [65]. The intensity and position of characteristic FTIR peaks were employed to evaluate the starch structure and protein secondary structure of millet fermented beverages (MFB) inoculated with different starter cultures (Figure 1B). The FTIR spectra of the fermented beverages were recorded in the range of 4000–400 cm^−1^ [66] (Figure 1B). No new characteristic functional groups were observed after fermentation, indicating that the controlled fermentation process did not generate new chemical groups [67].

To investigate the effects of different starter cultures on the protein components of the fermented millet beverages, the amide I band (1600–1700 cm^−1^), attributed to C=O stretching vibrations, was analyzed to evaluate changes in protein secondary structure [65]. As the amide II band is not suitable for quantitative analysis of protein secondary structure [65], the amide I region was selected for deconvolution and quantitative characterization using PeakFit v4.12 (Figure 1B). The following band assignments were applied: β-sheet (1600–1640 cm^−1^), random coil (1640–1650 cm^−1^), α-helix (1650–1660 cm^−1^), and β-turn (1660–1700 cm^−1^) [65]. As shown in Table 7, all MFB samples contained approximately 48–50% β-sheet, 20–23% random coil, and 29–30% β-turn structures, while no α-helix was detected. The secondary structure of MFB proteins was predicted and analyzed by Gaussian fitting of the amide I band extracted from the FTIR spectra [65].

Thermal processing, a commonly used physical treatment in the food industry, has long been studied for its effects on protein spatial conformation and functional properties [68]. In this study, the α-helix content was 0% in all fermented millet samples, indicating that the ordered helical structure was completely disrupted under the fermentation conditions employed, and this was highly correlated with the sample pretreatment. A similar reduction in α-helix content during heat treatment has also been reported [67], which is consistent with our observations. The sample preparation involved sterilization in a 100 °C water bath for 10 min, which may have caused the α-helix content to decrease to near zero. This is likely due to the gradual transition of proteins from initial conformational perturbation to irreversible aggregation during heating, manifested by a decrease in α-helix content and an increase in β-sheet structure [68].

In recent years, FTIR spectroscopy has been proposed to evaluate the short-range order of starch [69]. Previous studies [69] have shown that the FTIR spectra of starch in the 1100–900 cm^−1^ region are sensitive to changes in starch structure, particularly at the bands of 1000, 1022, and 1047 cm^−1^. This study has been widely studied by previous studies [70,71], so the band ratios of 1022/1000 cm^−1^ and 1047/1022 cm^−1^ were used in this study as a measure of the short-range ordered molecular structure. The higher the peak intensity ratio of 1047/1022 cm^−1^, the higher the order. The effect of lactic acid bacteria fermentation on the ordered structure of starch molecules increased with fermentation time, indicating progressive structural damage [65]. As shown in Table 7, the 1047/1022 cm^−1^ ratios of MFB-S, MFB-L, and MFB-2 were significantly lower than those of the control group (0.628–0.642). Lactic acid fermentation—particularly with dual-strain co-culture—erodes both the amorphous and crystalline regions of starch through its metabolites, disrupting the crystalline structure and weakening hydrogen bonds [72]. These structural changes promoted the depolymerization and molecular rearrangement of starch aggregates, ultimately resulting in a significant decrease in short-range starch order [73], which is consistent with previous findings [74]. Fermentation thus induces starch degradation and structural rearrangement, reducing the degree of short-range order. In contrast, the significantly higher ratios observed in the Bifidobacterium-containing groups (MFB-4, MFB-10, and MFB-12) may be attributed to their rapid metabolism and accelerated acidification [30], leading to more extensive destruction of the starch crystalline structure.

The 1022/1000 cm^−1^ ratio reflects the short-range order of double helices [75]. Regarding FTIR band assignment of starch, previous research [76] indicates that the strong absorption peak at 1000 cm^−1^ is clearly attributable to hydrated crystalline domains. A decrease in this peak suggests the disruption of hydrated crystalline domains. Among all samples, MFB-12 exhibited the highest 1022/1000 cm^−1^ ratio (11.5), indicating that this bacterial consortium induced the most pronounced changes in the short-range ordered structure of starch, which may be related to the synergistic co-metabolism of lactic acid bacteria and bifidobacteria.

In summary, single- or dual-strain fermentation disrupted the ordered structure of starch, whereas fermentation with four or more mixed strains (e.g., MFB-12) induced the formation of ordered structures, as reflected by increased characteristic band ratios. MFB-12 exhibited the most pronounced regulatory effect and may potentially generate functional starch complexes.

### 3.11. GC-MS Analysis of Fermented Millet Beverages Produced Using Commercial Fermenters of Different Strains

**Figure 3:**
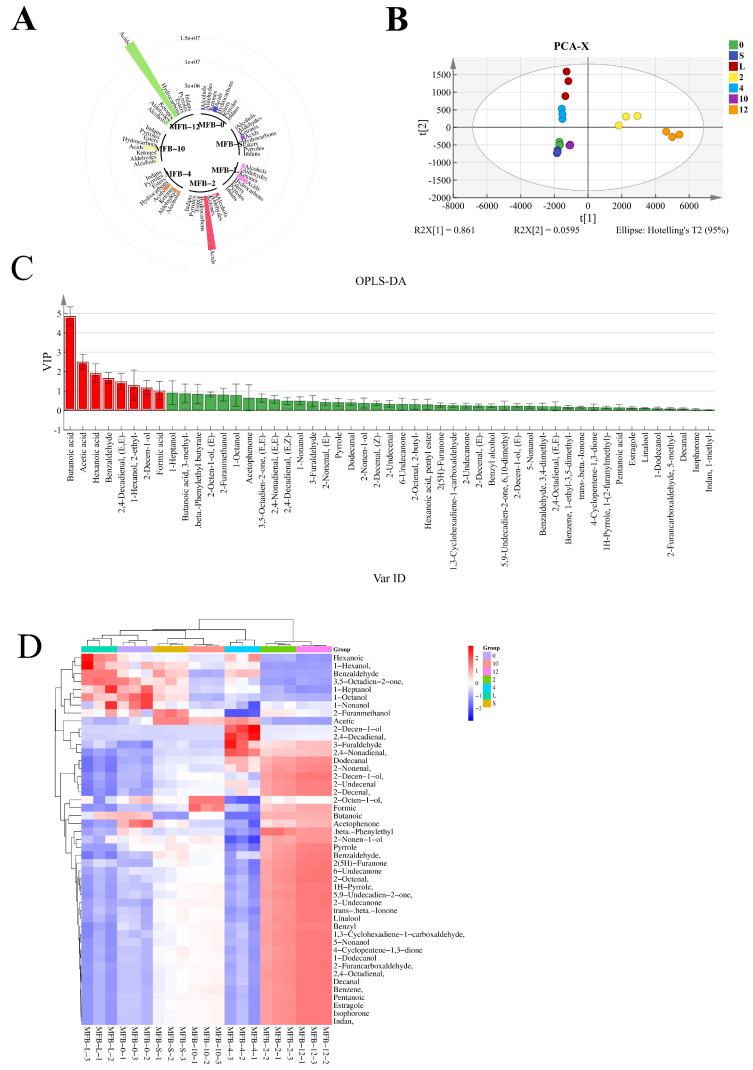
Circular bar chart (**A**), PCA plot (**B**), VIP plot (**C**), cluster heatmap (**D**). PCA: Principal Component Analysis, VIP: Variable Importance in Projection. Analysis results of volatile flavor compounds in millet fermented beverages inoculated with different lactic acid bacteria starters. MFB-0, MFB-S, MFB-L, MFB-2, MFB-4, MFB-10, and MFB-12 represent millet fermented beverage samples inoculated with *Streptococcus salivarius* subsp. *thermophilus*, *Lactobacillus delbrueckii* subsp. *bulgaricus*, 2 strains, 4 strains, 10 strains and 12 strains, respectively.

To characterize the volatile profiles of fermented millet beverages inoculated with different commercial starter cultures, headspace solid-phase microextraction coupled with gas chromatography–mass spectrometry (HS-SPME–GC–MS) was employed to identify and quantify volatile compounds. A total of 51 volatile compounds were detected (Table 8), comprising 13 alcohols, 16 aldehydes, 9 ketones, 6 carboxylic acids, 1 hydrocarbon, 2 esters, 2 indanes, and 2 pyrroles. The relative proportions of each chemical class are presented in Figure 3A. Carboxylic acids predominated, accounting for approximately 65% to 92% of the total volatile compounds across all samples, indicating their high abundance in the fermented beverages. As shown in Table 8, MFB-0 exhibited a baseline level of volatile compounds, although most were present at lower concentrations than those in the fermented groups. Previous studies [77] have reported correlations between lactic acid bacteria and volatile compound production during plant-based fermentation. Consistent with these reports [77], the volatile compounds in the fermented millet beverages are primarily derived from the raw materials and microbial metabolism during fermentation.

Principal component analysis (PCA) was performed based on the volatile compound data (Figure 3B). PC1 and PC2 accounted for 86.1% and 5.95% of the total variance, respectively, with a cumulative variance of 92.05%, indicating that the model effectively retained the original data information and achieved satisfactory sample discrimination [78]. The confidence ellipse (Hotelling’s T^2^ at 95%) showed partial overlap among sample groups, suggesting non-significant differences between some groups. MFB-2 and MFB-12 fell entirely within the right half of the ellipse, showing the greatest separation from the other groups, which suggests that their volatile profiles differed most substantially from the others; the co-culture group exhibited a richer profile of typical volatile compounds [38]. A clear trend of group separation was observed in the PCA score plot. MFB-12 was distinctly separated from the other groups along the PC1 axis, indicating that its volatile composition differed most significantly from the other treatments. MFB-2 also displayed distinct separation, whereas MFB-0, MFB-S, and MFB-10 were closely clustered, suggesting high similarity in their volatile compound compositions.

Orthogonal partial least squares discriminant analysis (OPLS-DA) with variable importance in projection (VIP) analysis further identified discriminant volatile compounds with high contribution (Figure 3C). Key compounds with VIP > 1 included butyric acid, acetic acid, caproic acid, benzaldehyde, 2,4-decadienal, 2-ethyl-1-hexanol, and 2-decen-1-ol, which served as core variables for distinguishing the volatile profiles among the different fermentation groups.

**Table 8:** Characterization of volatile flavor compounds in fermented millet beverages prepared with different lactic acid bacteria starters using GC-MS.

	MFB-0	MFB-S	MFB-L	MFB-2	MFB-4	MFB-10	MFB-12
1-Heptanol	70,794.06 ± 12,960.62 cd	43,147.88 ± 4150.63 d	157,781.08 ± 32,450.52 ab	188,067.13 ± 33,078.89 a	90,497.85 ± 805.34 c	75,259.28 ± 1426.95 cd	128,177.15 ± 2223.09 b
1-Hexanol, 2-ethyl-	66,706.00 ± 21,010.35 a	57,398.01 ± 5693.69 b	234,458.84 ± 86,342.36 b	83,231.75 ± 6254.53 b	118,782.89 ± 528.64 b	102,940.37 ± 11,604.59 b	108,099.76 ± 10,188.33 b
5-Nonanol	1234.13 ± 373.53 c	218.08 ± 47.09 d	923.57 ± 524.26 cd	1838.81 ± 213.76 bc	2630.60 ± 1009.65 ab	1052.58 ± 138.41 cd	3085.74 ± 516.84 a
Linalool	2379.82 ± 898.32 c	1893.69 ± 84.71 c	5630.92 ± 821.74 b	7010.76 ± 1412.60 b	3050.89 ± 1233.14 c	2509.64 ± 227.21 c	9631.81 ± 1313.46 a
1-Octanol	74,613.55 ± 10,220.07 c	36,554.62 ± 4311.22 d	125,484.78 ± 23,765.77 a	101,493.84 ± 14,823.49 b	57,483.14 ± 5120.39 c	35,767.17 ± 3522.63 d	74,789.98 ± 1249.58 c
2-Octen-1-ol, (E)-	25,754.68 ± 3913.72 cd	15,327.82 ± 864.08 d	39,750.04 ± 15,723.41 c	122,692.85 ± 18,079.47 a	12,469.30 ± 4332.13 d	73,070.34 ± 127.74 b	127,410.24 ± 4103.26 a
1-Nonanol	25,176.99 ± 3980.52 c	12,536.54 ± 1458.58 b	51,995.79 ± 9545.74 d	67,154.71 ± 9168.93 a	31,250.87 ± 5234.50 c	27,750.79 ± 1154.59 c	56,079.11 ± 853.99 b
2-Furanmethanol	18,710.66 ± 3803.21 de	19,342.18 ± 544.99 de	48,977.43 ± 4191.94 b	79,440.18 ± 160.57 a	14,775.27 ± 3243.81 e	30,404.53 ± 1036.00 cd	40,082.94 ± 16,219.49 bc
2-Nonen-1-ol	9761.72 ± 1895.36 de	4480.80 ± 785.36 ef	18,376.75 ± 7540.62 c	24,870.46 ± 3505.44 b	21.34 ± 6.67 f	12,194.88 ± 626.85 d	31,304.52 ± 3577.75 a
2-Decen-1-ol	5735.01 ± 852.99 b	1137.11 ± 200.10 b	13,998.85 ± 2853.25 b	8049.66 ± 319.37 b	215,078.26 ± 40,399.39 a	3607.96 ± 433.76 b	7913.92 ± 2648.96 b
2-Decen-1-ol, (E)-	452.40 ± 116.43 de	163.02 ± 18.13 e	1330.97 ± 202.20 bcd	1896.82 ± 171.24 b	9127.77 ± 1358.13 a	873.64 ± 82.51 cde	1727.72 ± 245.41 bc
Benzyl alcohol	4462.42 ± 1106.95 d	2232.86 ± 56.15 e	6618.03 ± 520.37 cd	15,720.48 ± 2548.71 a	7829.97 ± 1114.39 c	6622.46 ± 328.50 cd	13,241.34 ± 1373.70 b
1-Dodecanol	1063.55 ± 132.39 bc	1080.59 ± 49.58 bc	1322.13 ± 262.93 b	2007.27 ± 276.25 a	2225.53 ± 500.16 a	580.61 ± 62.73 c	1859.79 ± 456.59 a
3-Furaldehyde	2898.26 ± 1.95 c	3579.14 ± 644.62 c	20,618.45 ± 2351.62 b	6501.91 ± 2286.61 c	44,377.83 ± 11,525.09 a	3943.71 ± 142.82 c	8074.15 ± 2052.70 c
Decanal	15.84 ± 5.61 b	14.01 ± 1.31 b	35.56 ± 10.41 b	15.04 ± 3.05 b	1347.92 ± 439.37 a	15.68 ± 1.95 b	43.41 ± 4.03 b
Benzaldehyde	77,897.35 ± 14,694.00 de	97,685.79 ± 15,353.51 d	339,733.22 ± 39,546.00 a	96,942.71 ± 19,991.88 d	213,430.98 ± 18,548.94 b	53,424.84 ± 3219.14 e	154,995.57 ± 15,648.80 c
2-Nonenal, (E)-	3856.50 ± 443.51 de	2564.16 ± 230.68 de	6937.38 ± 503.08 c	13,162.42 ± 3813.32 b	23,857.48 ± 1639.46 a	1732.89 ± 201.33 e	5195.71 ± 212.63 cd
2-Furancarboxaldehyde, 5-methyl-	509.94 ± 226.60 d	411.03 ± 44.56 d	1963.07 ± 13.24 a	1251.50 ± 156.38 c	1854.48 ± 458.37 ab	337.49 ± 34.03 d	1501.81 ± 133.19 bc
2,4-Octadienal, (E,E)-	92.20 ± 14.41 b	149.75 ± 29.20 b	648.95 ± 42.19 b	2635.86 ± 1822.50 a	1314.11 ± 122.89 b	116.37 ± 11.78 b	412.05 ± 202.32 b
2-Decenal, (Z)-	3376.12 ± 2102.75 c	1929.33 ± 479.21 c	3748.41 ± 1157.47 c	12,579.30 ± 1735.94 b	23,150.70 ± 5366.76 a	3473.42 ± 252.28 c	9076.93 ± 1216.10 b
1,3-Cyclohexadiene-1-carboxaldehyde, 2,6,6-trimethyl-	1911.54 ± 1239.08 cd	867.15 ± 257.14 d	2375.10 ± 200.06 c	9221.25 ± 1081.54 a	4497.64 ± 256.83 b	2642.54 ± 138.63 c	5018.66 ± 46.44 b
2-Decenal, (E)-	627.52 ± 287.96 d	816.08 ± 168.81 d	3090.48 ± 507.90 c	6465.84 ± 1046.72 b	10,757.46 ± 1729.37 a	1604.64 ± 88.89 cd	5841.15 ± 1382.53 b
2-Octenal, 2-butyl-	2854.71 ± 1450.94 bc	516.02 ± 86.62 d	5598.26 ± 48.96 a	2424.18 ± 181.08 bc	3582.25 ± 665.26 b	1436.78 ± 238.36 cd	7132.24 ± 2086.24 a
2,4-Nonadienal, (E,E)-	1815.63 ± 898.44 c	1303.83 ± 320.91 c	3775.61 ± 204.17 c	12,579.58 ± 1805.64 b	48,427.70 ± 4197.85 a	538.31 ± 180.11 c	3023.19 ± 457.21 c
Dodecanal	1323.64 ± 126.16 c	933.48 ± 275.54 c	2343.27 ± 180.36 c	6472.66 ± 1337.87 b	25,587.36 ± 2042.75 a	892.58 ± 78.23 c	1624.48 ± 38.70 c
2-Undecenal	1108.87 ± 86.11 cd	150.05 ± 1.07 d	3134.53 ± 80.16 c	5739.28 ± 906.90 b	17,786.52 ± 3260.22 a	962.69 ± 54.19 cd	2524.27 ± 177.79 cd
2,4-Decadienal, (E,Z)-	875.26 ± 177.56 cd	29.50 ± 9.64 d	3797.86 ± 510.59 bc	5180.46 ± 524.43 b	38,784.81 ± 4079.71 a	1010.16 ± 143.68 cd	2117.56 ± 218.59 cd
2,4-Decadienal, (E,E)-	10,804.28 ± 2240.81 b	3650.85 ± 603.47 b	25,595.79 ± 4068.42 b	12,936.55 ± 2299.02 b	320,343.95 ± 39,896.16 a	5467.12 ± 1276.93 b	32,045.77 ± 12,477.98 b
Benzaldehyde, 3,4-dimethyl-	3821.47 ± 897.66 b	3974.64 ± 947.81 b	5491.40 ± 122.68 b	4293.68 ± 65.08 b	10,878.70 ± 4671.14 a	3462.39 ± 184.56 b	5986.44 ± 298.96 b
6-Undecanone	3837.58 ± 1259.21 b	220.77 ± 57.56 c	5642.92 ± 655.04 a	260.44 ± 25.52 c	274.08 ± 21.83 c	118.00 ± 28.29 c	148.69 ± 45.43 c
3,5-Octadien-2-one, (E,E)-	35,941.28 ± 4970.89 de	23,783.62 ± 1175.51 e	95,652.71 ± 10,983.35 a	50,397.48 ± 9172.79 bcd	53,220.97 ± 5727.80 bc	40,404.93 ± 643.51 cd	61,134.74 ± 13,537.11 b
4-Cyclopentene-1,3-dione	2.19 ± 2.69 b	915.66 ± 161.84 b	2.04 ± 0.07 b	2.40 ± 0.99 b	2.01 ± 0.85 b	3.65 ± 0.33 b	3697.86 ± 2043.03 a
Isophorone	215.92 ± 68.39 bcd	124.33 ± 21.94 d	345.68 ± 66.58 abc	437.43 ± 209.52 a	186.24 ± 86.22 cd	186.79 ± 13.10 cd	373.63 ± 60.92 ab
2-Undecanone	57.28 ± 5.39 d	1423.08 ± 183.05 c	4855.18 ± 759.44 a	73.33 ± 9.96 d	2540.22 ± 581.33 b	2613.45 ± 145.87 b	2434.12 ± 1267.17 bc
Acetophenone	19,568.33 ± 2942.86 b	7625.32 ± 1162.13 d	19,721.28 ± 235.49 b	19,404.19 ± 2163.92 b	14,837.82 ± 3055.11 c	10,604.98 ± 339.05 d	29,453.77 ± 1374.23 a
2(5 H)-Furanone	1740.30 ± 794.44 e	2292.40 ± 133.27 de	13,075.09 ± 466.70 a	7069.62 ± 41.38 c	2970.08 ± 917.80 d	1706.32 ± 87.45 e	9470.10 ± 1156.86 b
5,9-Undecadien-2-one, 6,10-dimethyl-, (E)-	1955.65 ± 516.02 c	148.08 ± 63.33 e	2781.14 ± 4.64 ab	3117.83 ± 378.37 a	1990.68 ± 480.78 c	1282.03 ± 11.73 d	2338.18 ± 441.07 bc
trans-.beta.-Ionone	2161.11 ± 503.26 cd	1772.37 ± 280.55 d	7233.75 ± 40.99 a	4880.03 ± 275.27 b	1868.05 ± 610.07 d	2629.55 ± 0.80 c	4322.56 ± 632.89 b
Acetic acid	278,285.45 ± 68,560.46 e	567,696.63 ± 40,609.40 d	381,396.60 ± 68,439.06 de	1,767,964.18 ± 228,192.32 b	1,137,217.01 ± 132,638.72 c	1,234,669.88 ± 27,395.54 c	2,320,889.72 ± 258,095.74 a
Formic acid	3579.38 ± 257.98 bc	1487.32 ± 197.56 c	4521.14 ± 1910.48 bc	4470.39 ± 1147.69 bc	8221.45 ± 3713.27 b	33,106.65 ± 5190.49 a	5082.67 ± 1114.20 bc
Butanoic acid	604,784.40 ± 25,702.95 c	126,481.76 ± 2189.30 c	1,066,948.91 ± 280,961.83 c	8,597,188.75 ± 1,375,059.55 b	154,499.48 ± 2329.46 c	1,144,753.81 ± 6677.93 c	15,004,523.48 ± 712,472.01 a
Butanoic acid, 3-methyl-	53,388.59 ± 6570.52 b	22,005.64 ± 299.21 c	157,681.35 ± 36,366.73 a	6929.92 ± 1060.40 c	1765.44 ± 370.24 c	3526.93 ± 50.39 c	5532.09 ± 1405.13 c
Pentanoic acid	1290.93 ± 149.56 d	1004.45 ± 249.70 d	2808.46 ± 223.77 bc	5442.98 ± 1141.10 a	1692.44 ± 273.64 cd	2119.41 ± 271.27 cd	3889.27 ± 1429.83 b
Hexanoic acid	220,637.01 ± 32,955.05 cd	160,937.12 ± 6730.73 d	914,932.69 ± 226,768.87 a	999,094.39 ± 193,197.12 a	561,264.39 ± 112,910.56 b	405,171.91 ± 10,465.08 bc	857,320.93 ± 47,518.08 a
Estragole	514.50 ± 42.10 cd	575.57 ± 128.98 cd	885.74 ± 68.02 c	2007.79 ± 225.88 a	577.66 ± 109.66 b	371.67 ± 100.93 bc	1371.39 ± 626.45 a
Hexanoic acid, pentyl ester	25.35 ± 9.46 c	2496.48 ± 600.44 a	55.65 ± 15.98 c	19.48 ± 10.18 c	14.05 ± 3.66 c	1558.83 ± 833.30 b	26.90 ± 0.26 c
.beta.-Phenylethyl butyrate	10,715.60 ± 2570.78 c	2063.36 ± 453.29 d	10,843.38 ± 367.12 c	95,266.63 ± 9955.60 a	4334.43 ± 499.92 cd	4572.61 ± 241.64 cd	65,327.65 ± 1827.07 b
Pyrrole	7671.92 ± 1635.75 b	4291.64 ± 19.51 d	9933.63 ± 2766.35 b	9075.36 ± 936.31 b	4597.49 ± 577.19 cd	7318.35 ± 200.09 bc	18,996.37 ± 2468.91 a
1 H-Pyrrole, 1-(2-furanylmethyl)-	1895.50 ± 548.14 c	1135.10 ± 133.59 d	4273.43 ± 428.72 a	2617.26 ± 230.71 bc	2084.81 ± 306.32 bc	2128.33 ± 146.56 bc	2845.37 ± 686.98 b
Benzene, 1-ethyl-3,5-dimethyl-	0.79 ± 0.23 b	1.13 ± 0.65 b	2.86 ± 1.30 b	1.06 ± 0.52 b	1.76 ± 1.30 b	1181.47 ± 168.20 a	2.70 ± 0.07 b
Indan, 1-methyl-	18.38 ± 5.62 cd	17.35 ± 11.25 cd	52.06 ± 14.81 a	25.94 ± 3.64 bc	18.22 ± 3.96 cd	9.18 ± 1.74 d	35.90 ± 4.81 b

Note: Values are means ± STD (*n* = 3). Values with different letters in the same column are significantly different (*p* < 0.05). MFB-0, MFB-S, MFB-L, MFB-2, MFB-4, MFB-10, and MFB-12 represent millet fermented beverage samples inoculated with *Streptococcus salivarius* subsp. *thermophilus*, *Lactobacillus delbrueckii* subsp. *bulgaricus*, 2 strains, 4 strains, 10 strains and 12 strains, respectively.

During fermentation, microorganisms produce various alcohols through glycolysis, amino acid decarboxylation, and dehydrogenation. These alcohols not only contribute directly to the volatile profile but also serve as key precursors for ester synthesis [79]. Based on VIP values (>1) from the OPLS-DA model (Figure 3C) and intergroup difference analysis, 2-ethyl-1-hexanol and 2-decen-1-ol were identified as the key discriminant alcohols. 2-Ethyl-1-hexanol was significantly enriched in the mixed fermentation groups (especially MFB-12 and MFB-2), contributing to volatile complexity while also serving as an important precursor for ester synthesis [78], acting synergistically with aldehydes, ketones, and other volatiles to form a complex volatile system. A previous study [80] identified 2-decen-1-ol as one of the primary compounds distinguishing regional differences among fermented samples; thus, variations in its concentration may serve as a key variable differentiating mono- from poly-microbial fermentation profiles. 2-Decen-1-ol was significantly enriched in MFB-12 and also present at high levels in MFB-2, possibly because multi-microbial synergistic fermentation, through metabolic complementarity among strains, increases the total yield of alcohols and the abundance of specific compounds [81].

As shown in Figure 3A, carboxylic acids were the predominant volatile components. They originate from lipid or protein hydrolysis [82], and are associated with the metabolic pathways of lactic acid bacteria. Among the fatty acids detected, caproic acid and butyric acid exhibited significantly higher relative contents than other fatty acids (*p* < 0.05) (Table 8). Acetic acid levels were significantly elevated in the multi-strain fermentation groups (especially MFB-12) and acted in synergy with other compounds such as alcohols and aldehydes. Furthermore, carboxylic acids serve as precursors to ketones, aldehydes, alcohols, and esters, all of which may influence the volatile profile of the fermented beverages [83]. Aldehydes represented the largest chemical class and constituted the primary volatile components. Benzaldehyde was detected at higher concentrations in MFB-L and MFB-12, whereas its content was extremely low in MFB-0 and MFB-S, suggesting that multi-microbial co-fermentation may promote aldehyde formation. As shown in Figure 3D, 2,4-decadienal was significantly highest in MFB-4 and also showed relatively high concentrations in MFB-12, while its content was extremely low in MFB-0, MFB-S, and MFB-L.

Overall, MFB-12 exhibited the greatest diversity of volatile compounds, with acids and alcohols as the predominant classes. Although aldehydes and other compounds were present in lower relative abundances, their contributions to the overall volatile profile, in combination with the major components, resulted in the most complex volatile fingerprint among all samples, which is consistent with the sensory evaluation results (Section 3.12), where MFB-12 received the highest scores for aroma and overall acceptability (Table 9).

### 3.12. Sensory Evaluation

The sensory evaluation results of millet fermented beverages inoculated with different types of starter cultures are presented in Table 9. The overall sensory attribute scores of all fermented beverages were within their acceptable range (4–9), as described in the sensory evaluation section. As shown in Table 9, all samples exhibited good performance in appearance and mouthfeel, with scores above 5.00, ranging from 6.40 to 7.00 for appearance and 5.67 to 6.40 for mouthfeel. No significant differences were observed among groups (*p* > 0.05), indicating that the type of starter culture had little influence on the physical properties such as color, organizational state, and smoothness of the millet fermented beverages. In terms of aroma and taste, considerable variations were observed among samples. MFB-12 received the highest scores for both aroma (6.73) and taste (6.53), which were significantly higher than those of the other groups (*p* < 0.05). In contrast, MFB-2 and MFB-4 scored below 5 for both aroma and taste, suggesting that fermentation with certain strains resulted in poor flavor quality. Regarding overall acceptability, only MFB-12 achieved a score above 5 (6.40), which was significantly superior to the other samples (*p* < 0.05). The remaining samples scored between 4.80 and 5.53, with MFB-2 and MFB-4 falling below the cutoff score of 5, indicating low consumer acceptance. In conclusion, the type of starter culture significantly influenced the aroma and taste of millet fermented beverages, while having limited effects on appearance and mouthfeel. Among all samples, MFB-12 exhibited the best performance in improving the sensory quality of millet fermented beverages.

## 4. Conclusions

This study systematically evaluated the effects of single- and multi-strain lactic acid bacteria starters on the quality attributes of fermented millet beverages. The primary novelty resides in the systematic comparison across two single strains and four multi-strain consortia comprising 2, 4, 10, and 12 strains, which revealed that incremental increases in microbial complexity exert divergent influences on structural versus sensory properties. MFB-2 exhibited the highest viscosity and gel strength, whereas MFB-10 and MFB-12 demonstrated superior gel stability, water-holding capacity, and more harmoniously balanced volatile profiles. Notably, the 10-strain consortium attained structural stability comparable to that of the 12-strain formulation, while exhibiting milder acidity and a smoother mouthfeel, thereby challenging the prevailing assumption that greater microbial diversity is unequivocally advantageous. From an industrial standpoint, MFB-2 is recommended for thick-set, spoonable products, whereas MFB-10 and MFB-12 are better suited for drinkable, shelf-stable beverage applications. Future investigations should focus on elucidating the metabolic interactions among constituent strains and validating the scalability of these formulations through pilot-scale production trials. Collectively, this study furnishes a scientific foundation for the rational design of starter culture complexity in the development of high-quality plant-based beverage alternatives.

## Figures and Tables

**Table 1:** Basic nutritional components of millet fermented beverages inoculated with different lactic acid bacteria starters.

Sample	Protein (%)	Fat (%)	Total Starch (mg/100 mL)	Carotenoids (mg/kg)
MFB-0	0.9420 ± 0.0037 b	0.70 ± 0.12 a	54.46 ± 0.81 c	21.11 ± 0.37 b
MFB-S	0.7653 ± 0.0064 d	0.56 ± 0.12 b	56.67 ± 3.94 c	18.52 ± 0.00 c
MFB-L	0.7670 ± 0.0026 d	0.58 ± 0.11 ab	54.37 ± 0.47 c	22.72 ± 0.21 a
MFB-2	0.7904 ± 0.0045 c	0.25 ± 0.01 c	69.50 ± 0.32 a	15.17 ± 1.62 d
MFB-4	0.9589 ± 0.0049 a	0.26 ± 0.03 c	65.95 ± 0.18 b	14.81 ± 0.98 d
MFB-10	0.9531 ± 0.0043 a	0.33 ± 0.03 c	70.06 ± 0.84 a	16.05 ± 0.57 d
MFB-12	0.7866 ± 0.0033 c	0.51 ± 0.02 b	66.74 ± 0.05 b	10.62 ± 0.42 e

Note: Values are means ± STD (*n* = 3). Values with different letters in the same column are significantly different (*p* < 0.05). MFB-0, MFB-S, MFB-L, MFB-2, MFB-4, MFB-10, and MFB-12 represent millet fermented beverage samples inoculated with *Streptococcus salivarius* subsp. *thermophilus*, *Lactobacillus delbrueckii* subsp. *bulgaricus*, 2 strains, 4 strains, 10 strains and 12 strains, respectively.

**Table 2:** Water holding capacity (WHC) and pH values of millet fermented beverages with different lactic acid bacteria starters.

Sample	WHC (%)	PH
MFB-0	94.89 ± 2.57 a	4.60 ± 0.06 a
MFB-S	93.93 ± 0.20 ab	3.91 ± 0.05 c
MFB-L	93.51 ± 1.81 ab	4.03 ± 0.02 b
MFB-2	91.35 ± 1.02 b	3.69 ± 0.02 d
MFB-4	92.17 ± 2.78 ab	3.32 ± 0.01 d
MFB-10	92.17 ± 1.33 ab	3.50 ± 0.05 e
MFB-12	95.56 ± 0.95 a	3.55 ± 0.01 e

Note: Values are means ± STD (n = 3). Values with different letters in the same column are significantly different (*p* < 0.05). MFB-0, MFB-S, MFB-L, MFB-2, MFB-4, MFB-10, and MFB-12 represent millet fermented beverage samples inoculated with *Streptococcus salivarius* subsp. *thermophilus*, *Lactobacillus delbrueckii* subsp. *bulgaricus*, 2 strains, 4 strains, 10 strains and 12 strains, respectively.

**Table 4:** Analysis of rheological properties of millet fermented beverages inoculated with different lactic acid bacteria starters.

Category	Peak Viscosity (cP)	Trough Viscosity (cP)	Breakdown (cP)	Final Viscosity (cP)	Setback
MFB-0	211.3 ± 7.11 c	53.61 ± 1.85 b	157.70 ± 8.84 a	138.97 ± 2.48 c	85.35 ± 4.28 c
MFB-S	194.33 ± 4.16 ab	57.24 ± 0.55 a	137.10 ± 3.68 c	150.53 ± 2.54 b	93.29 ± 2.31 b
MFB-L	193.13 ± 2.47 a	57.59 ± 0.82 a	138.53 ± 1.88 bc	153.03 ± 1.01 b	95.44 ± 0.50 ab
MFB-2	194.57 ± 7.46 ab	46.94 ± 0.82 c	147.63 ± 6.84 b	123.83 ± 2.01 d	76.90 ± 1.26 d
MFB-4	185.4 ± 4.64 b	58.55 ± 0.82 a	126.87 ± 4.71 d	156.40 ± 1.71 a	97.85 ± 1.39 a
MFB-10	160.87 ± 2.29 c	42.45 ± 1.37 d	118.47 ± 1.21 d	105.67 ± 0.64 e	63.22 ± 1.86 d
MFB-12	156.6 ± 5.24 c	29.83 ± 0.66 e	126.77 ± 5.23 d	75.69 ± 1.45 f	45.86 ± 1.48 e

Note: Values are means ± STD (*n* = 3). Values with different letters in the same column are significantly different (*p* < 0.05). MFB-0, MFB-S, MFB-L, MFB-2, MFB-4, MFB-10, and MFB-12 represent millet fermented beverage samples inoculated with *Streptococcus salivarius* subsp. *thermophilus*, *Lactobacillus delbrueckii* subsp. *bulgaricus*, 2 strains, 4 strains, 10 strains and 12 strains, respectively.

**Table 7:** The relative composition of protein secondary structure and the ratio of absorbance of starch fingerprint region 1047/1022 cm^−1^ and 1022/1000 cm^−1^ of millet fermented beverages inoculated with different lactic acid bacteria starters.

Samples	β-Sheet (%)	Random Coil (%)	α-Helix (%)	β-Turn (%)	1047/1022 cm^−1^	1022/1000 cm^−1^
MFB-0	49.83 ± 0.06 a	20.64 ± 0.04 b	-	29.54 ± 0.07 ab	0.658 ± 0.014 bc	3.686 ± 0.296 cd
MFB-S	49.81 ± 0.05 a	20.65 ± 0.05 b	-	29.54 ± 0.07 ab	0.628 ± 0.010 c	4.750 ± 1.624 c
MFB-L	49.74 ± 0.06 a	20.67 ± 0.01 b	-	29.59 ± 0.06 ab	0.628 ± 0.014 c	3.743 ± 0.072 cd
MFB-2	48.58 ± 2.20 a	21.61 ± 1.66 ab	-	29.81 ± 0.54 a	0.642 ± 0.003 c	3.443 ± 0.047 d
MFB-4	48.12 ± 0.03 a	22.70 ± 0.01 a	-	29.19 ± 0.03 b	0.702 ± 0.003 a	10.056 ± 0.096 b
MFB-10	49.79 ± 0.02 a	20.63 ± 0.01 b	-	29.58 ± 0.02 ab	0.704 ± 0.009 a	9.944 ± 0.347 b
MFB-12	49.20 ± 1.00 a	21.36 ± 1.19 ab	-	29.44 ± 0.19 ab	0.688 ± 0.050 ab	11.500 ± 0.667 a

Note: Values are means ± STD (*n* = 3). Values with different letters in the same column are significantly different (*p* < 0.05). MFB-0, MFB-S, MFB-L, MFB-2, MFB-4, MFB-10, and MFB-12 represent millet fermented beverage samples inoculated with *Streptococcus salivarius* subsp. *thermophilus*, *Lactobacillus delbrueckii* subsp. *bulgaricus*, 2 strains, 4 strains, 10 strains and 12 strains, respectively.

**Table 9:** Sensory evaluation results of millet fermented beverages inoculated with different types of starter cultures.

Sample	Appearance	Aroma	Taste	Mouthfeel	Overall Acceptability
MFB-0	6.40 ± 0.63 b	5.33 ± 1.18 b	5.20 ± 1.37 bc	6.00 ± 1.46 a	5.47 ± 0.99 b
MFB-S	6.60 ± 0.63 ab	4.87 ± 1.19 b	5.27 ± 1.16 bc	5.67 ± 1.50 a	5.33 ± 1.05 b
MFB-L	6.67 ± 0.72 ab	5.27 ± 1.03 b	5.47 ± 0.92 b	6.00 ± 1.36 a	5.40 ± 0.99 b
MFB-2	6.40 ± 0.74 b	4.53 ± 0.83 b	4.73 ± 0.96 bc	6.07 ± 1.33 a	4.80 ± 1.01 b
MFB-4	6.60 ± 0.51 ab	4.60 ± 1.24 b	4.47 ± 1.36 c	6.13 ± 1.13 a	4.87 ± 1.06 b
MFB-10	6.53 ± 0.74 ab	5.27 ± 0.88 b	5.20 ± 1.37 bc	6.40 ± 1.18 a	5.53 ± 0.99 b
MFB-12	7.00 ± 0.53 a	6.73 ± 0.96 a	6.53 ± 0.99 a	6.13 ± 0.92 a	6.40 ± 0.63 a

Note: Values are means ± STD (*n* = 3). Values with different letters in the same column are significantly different (*p* < 0.05). MFB-0, MFB-S, MFB-L, MFB-2, MFB-4, MFB-10, and MFB-12 represent millet fermented beverage samples inoculated with *Streptococcus salivarius* subsp. *thermophilus*, *Lactobacillus delbrueckii* subsp. *bulgaricus*, 2 strains, 4 strains, 10 strains and 12 strains, respectively.

## Data Availability

The original contributions presented in the study are included in the article; further inquiries can be directed to the corresponding author.
